# Identification and characterization of a heat-inducible Hsp70 gene from S*orghum bicolor* which confers tolerance to thermal stress

**DOI:** 10.1007/s12192-015-0591-2

**Published:** 2015-06-14

**Authors:** Takalani Mulaudzi-Masuku, Rendani Daphney Mutepe, Ofhani Christopher Mukhoro, Andrew Faro, Bongani Ndimba

**Affiliations:** Proteomics Research and Services Unit, Department of Biotechnology, University of the Western Cape, Level 2—New Life Sciences Building, Modderdam Road, Private Bag X17, Bellville, 7535 Cape Town South Africa; Proteomics Unit, Agricultural Research Council, Infruitec-Nietvoorbij, Private Bag X5026, Stellenbosch, 7599 South Africa

**Keywords:** ATPase assay, Cloning, Gene expression, Heat shock protein 70 (Hsp70), *Sorghum bicolor*, SbHsp70-1, Thermotolerance

## Abstract

This study describes the first detailed molecular characterization of the heat shock protein 70 (Hsp70) gene from *Sorghum bicolor*, MN1618 designated as SbHsp70-1. The full-length cDNA of SbHsp70-1 consists of 2524 bp with a 1950 bp open reading frame, which encodes a protein of 649 amino acids. SbHsp70-1 is a cytoplasmic protein with high homology to other plant Hsp70s, especially grain crops. Recombinant SbHsp70-1 was able to bind and hydrolyse ATP in a dose-dependent manner, suggesting that SbHsp70-1 functions as an ATPase. Immunoblot assays showed that the expression of SbHsp70-1 is induced at temperatures of 37, 45, and 4 °C but reduced at 42 °C. In addition, the SbHsp70-1 mRNA transcript is constitutively expressed in both leaves and stem but is significantly increased upon heat shock at 42 °C. Upon cold shock at 4 °C, SbHsp70-1 mRNA transcript level increased in the leaf, but no significant change was observed in the stem. In addition, expression of the pET28a-SbHsp70-1 construct in *Escherichia coli* cells under heat stress resulted in their survival even at higher temperature (65 °C). Our results suggest that SbHsp70-1 is a heat-inducible protein that confer thermal tolerance to bacterial cells and can be claimed as a promising target to study stress tolerance in crops.

**Electronic supplementary material:**

The online version of this article (doi:10.1007/s12192-015-0591-2) contains supplementary material, which is available to authorized users.

## Introduction

Plants are constantly exposed to abiotic and biotic stresses, with abiotic stress being the major limiting factor of crop growth and productivity worldwide (Mahajan and Tuteja 2005). Abiotic stresses have affected agricultural productivity, leading to more than 50 % crop loss (Wang et al. 2003). Stressed plants undergo various physiological and metabolic changes. However, they have evolved a wide range of mechanisms such as the induction of heat shock proteins (Hsps) to adapt to stressful conditions (Boston et al. 1996; Waters et al. 1996). Hsps are a class of ubiquitous and highly conserved proteins which are expressed in response to temperature or other environmental stresses (de Maio 1999). They are also constitutively present in normal or stressed cells. Hsps function as molecular chaperones in a variety of cellular processes including protein folding, protein transport across cellular membranes, modulation of protein activity, regulation of protein degradation, and prevention of irreversible protein aggregation (Vierling 1991). In eukaryotes, Hsps are normally classified according to their molecular weight as Hsp90, Hsp70, Hsp60, and small heat shock protein families. The 70-kDa heat shock proteins (Hsp70s) are the most abundant and widely studied conserved family of proteins. Hsp70 genes are encoded by a major Hsp multi-gene family made up of the cytosolic Hsp70s including the inducible Hsp70 and the cognate Hsc70, glucose-regulated protein 78 (Grp78), and the mitochondrial Hsp70 (Sung et al. 2001; Gething and Sambrook 1992; Ellis 1987). Hsp70 is characterized by two functional domains, the amino (N)-terminal ATPase domain (44 kDa) exhibiting an ATPase activity and a carboxyl (C)-terminal peptide-binding domain (25 kDa). The peptide-binding domain is further subdivided into a β-sandwich subdomain (18 kDa), which is the substrate-binding domain and an α-helical subdomain (Flaherty et al. 1990; Zhu et al. 1996; Wang et al. 1993). Hsp70s perform their roles due to their ability to interact with hydrophobic peptide segments of proteins in an ATP-dependent manner (Mayer and Bukau 2005). Depending on the ATP cycle, Hsp70 binds to the substrate peptide of unfolded or denatured proteins at its either normal or stress state (Szabo et al. 1994; Bukau and Horwich 1998). Hsp70s are important in developmental processes, and they function in various stresses including heat, cold, water deficit, and oxidative stress, among others (Heikkila et al. 1984; Dhankher et al. 1997; Chong et al. 1998). Under heat stress, a heat shock response is initiated by up-regulating the expression of Hsps (Lindquist and Craig 1988). Several heat-inducible Hsp70s have been reported previously to have a role in heat tolerance in various plant species including *Nicotiana tabacum* (Cho and Hong 2004), *Triticum aestivum* (Duan et al. 2011), *Dactylis glomerata* (Cha et al. 2012), *Oryza sativa* (Sarkar et al. 2013), and *Capsicum annuum* (Guo et al. 2014). Overexpression of the Arabidopsis cytosolic Hsc70-1 resulted in enhanced heat tolerance under certain conditions (Sung and Guy 2003). In *Arabidopsis thaliana*, the presence of a chrysanthemum CgHsp70 enhanced tolerance to abiotic stress, thus protecting the plants from total damage (Song et al. 2014).

Despite the breakthroughs in the identification and characterization of plant Hsps, in sorghum, the most widely cultivated and stress-tolerant cereal crop in the world (Krishnamurthy et al. 2007), the mechanism of stress tolerance through Hsp70 has never been reported before. Due to the effect of various stresses on plants, investigating various mechanisms of plant stress responses is crucial. Thus, the identification and characterization of Hsp70 genes in sorghum will aid in a better understanding of the molecular mechanism of its stress tolerance. In sorghum, the existence of Hsp70 was verified by immunoblotting following salt stress (Ndimba et al. 2010; Ngara et al. 2012). However, there are no studies describing the Hsp70 gene and its molecular characterization in sorghum. In this study, an Hsp70 gene was isolated and cloned from a sweet sorghum cultivar “MN1618” designated as SbHsp70-1. The nucleotide and the deduced amino acid sequences were analyzed using bioinformatics tools. SbHsp70-1 was recombinantly expressed and demonstrated to have a functional ATPase domain. In addition, the expression patterns of SbHsp70-1 at both the protein and mRNA level were investigated in response to various temperature stresses. Finally, its chaperone role was investigated by overexpressing a pET28a-SbHsp70-1 construct in *Escherichia coli* cells under heat stress, and SbHsp70-1 was able to confer heat tolerance to cells.

## Materials and methods

### Plant growth and treatment

Sorghum seedlings were germinated as previously described (Ngara et al. 2012). Sweet sorghum (*S. bicolor*, MN1618 cultivar) seeds were obtained from Dr. Pangirai Tongoona, University of KwaZulu-Natal, Pietermaritzburg, South Africa. The seeds were surface-sterilized with 70 % (*v*/*v*) ethanol for a minute followed by 20 % sodium hypochlorite solution for 20 min. The seeds were planted in 175 ml capacity plant tissue culture vessels (Sigma-Alrich, Saint Louis, MO, USA) containing 50 ml of half-strength MS media [2.2 g/l Murashige and Skoog basal medium (MS); 1 % (*w*/*v*) sucrose; 5 mM MES and 0.8 % (*w*/*v*) agar, pH 5.8]. The culture vessels were incubated at 25 °C under a 16 h light/8 h dark regime for 14 days. For temperature treatments, seedlings were treated in culture vessels by incubating them at different temperatures (37, 42, 45, and 4 °C) for 2 h. Following temperature treatments, seedling shoots were immediately frozen in liquid nitrogen and stored at −80 °C until further use. All treatments were conducted in triplicate.

### Total RNA extraction and reverse transcriptions

Total RNA was extracted from 100 mg of 2-week-old whole seedlings using the RNeasy Plant Mini Kit (Qiagen GmbH, Germany) according to the manufacturer’s instructions. After treatment with RNase-free DNase Set (Qiagen GmbH, Germany) to remove genomic DNA, 1 μg of the total RNA was used for first-strand cDNA synthesis using the cDNA reverse transcriptase kit (Roche Applied science, Germany) according to the manufacturer’s instructions, using anchored and random primers.

### Cloning of SbHsp70-1 and construction of expression vector

Polymerase chain reaction (PCR) was used to obtain the sorghum Hsp70 cDNA using a combination of forward SbHsp70-1fwd (5′-ggt*ggatcc*atggccggaaagggagac-3′) and reverse SbHsp70-1rev (5′-ggt*ctcgag*tgattagtcgacttcttcgatctt-3′) primers. The italicized sequence denotes the restriction enzyme recognition sequences for *Bam*HI and *Xho*I in the forward and reverse primers, respectively. Primers for the cDNA were designed based on the phytozome sequence (Phytozome version 9.1 in the *S. bicolor* database version 1.4) annotated as hypothetical protein, GeneBank accession no. XM_002468052.1; GI: 242041404. In the PCR, initial denaturation was done for 1 min at 96 °C, followed by 35 cycles under the following conditions: denaturation for 30 s at 94 °C, primer annealing for 1 min at 54 °C, DNA strand extension for 2 min at 72 °C using Phusion High Fidelity DNA polymerase (Thermo Scientific, #F-534 L, USA). The 35 cycles were followed by a final extension step at 72 °C for 10 min. The amplified cDNA fragments were cut out from the agarose gel and purified using the GeneJet gel purification kit (Fermentas Life Sciences, K#0692) and cloned into pET28a vector previously digested with the same enzymes as the insert to form the pET28a-SbHsp70-1 construct and sequenced within the pET28a. To verify the full-length cDNA after cloning, the pET28a construct was re-amplified using the same PCR conditions as above followed by a double digest to release the insert. The construct was also verified by sequencing done at Inqaba laboratories (Inqaba Biotechnical Industries (Pty) Ltd, South Africa).

### Sequence analysis

The cDNA sequences were analyzed using Blast and open reading frame (ORF) finder. Protein sequences were analyzed using Compute pI/Mw tool to compute the theoretical isoelectric point (pI) and protein molecular weight (Mw). InterPro-Scan and PROSITE scan were used for the prediction of signal peptides, conserved domains, and motifs, NetPhosK 1.0 for kinase-specific phosphorylation site prediction, YLoc for the prediction of subcellular localization, and ClustalX and MEGA 6.0 program (Tamura et al. 2013 ) for the sequence alignment and construction of a phylogenetic tree, respectively. All bioinformatics tools were obtained from the Expasy bioinformatics resource portal (http://www.expasy.org/tools/) unless stated otherwise.

### Expression and purification of the recombinant SbHsp70-1

Single colonies of *E. coli* BL21-CodonPlus® competent cells transformed with pET28a-SbHsp70-1 expression plasmid were inoculated into Luria–Bertani (LB) supplemented with 0.4 % glucose and 50 μg/ml kanamycin and incubated at 37 °C, with shaking overnight. The overnight culture was diluted 1:10 with fresh LB and grown until the cells reached an optical density of 0.5 at 600 nm (OD_600_). Induction of protein expression was done by the addition of isopropyl-1-thio-d-galactopyranoside (IPTG) to a final concentration of 1 mM for 4 h at 30 °C and harvested by centrifugation at 3,500×*g* for 10 min at 4 °C. The pellet was re-suspended in native lysis buffer (50 mM Na-phosphate, pH 8.0; 300 mM NaCl; 40 mM imidazole; 0.1 % Triton X-100; 50 μg/ml lysozyme; Protease inhibitor cocktail), and the cells were lysed by sonication and the cellular debris collected by centrifugation at 3,500×*g* for 30 min at 4 °C. The protein was purified as a carboxyl-terminal fusion to Histidine using Ni-NTA beads (Sigma-Aldrich, St. Louis, MO, USA) under native conditions. Two 5-ml fractions were collected, and the presence of eluted proteins was confirmed by analyzing the fractions on a 12 % SDS-PAGE. Fractions corresponding to SbHsp70-1 fusion protein were pooled and concentrated using Macrosep® Advance centrifugal devices (3 k MWCO; Pall Corporation, Separation. Solutions, USA). Protein concentrations were determined using the Bradford assay method (Bradford 1976).

### ATPase assays

ATPase activity was examined by measuring concentrations of released phosphate (PO_4_) using a modified malachite green colorimetric assay based on the ATPase assay previously described (Chan et al. 1986). Prior to ATPase assay analysis, the recombinant protein was dialysed overnight against 1X TEA buffer (50 mM Triethanolamine-HCl, 50 mM KCl, 20 mM MgCl_2_, pH 7.5). The assay buffer (0.033 % malachite green, 1.029 % ammonium molybdate, and 0.02 % Triton X-100) was incubated at room temperature for 1 h, after which it was filtered using a 0.45 μm filter and incubated for a further 1 h before use. A reaction was composed of 20 μM SbHsp70-1 recombinant protein (for the ATPase activity measurements), water (for determination of the natural hydrolysis of ATP) or KH_2_PO_4_ (for standard curve determination), 5 mM DTT, 1X TEA buffer, and 800 μl assay dye in the presence or absence of 5 mM adenosine triphosphate (ATP) as substrate, incubated at 25 °C for 1 min and supplemented with 100 μl of 34 % citric acid. To monitor the effect of time on the activity of SbHsp70-1, the ATPase assays were conducted in reactions which were subsequently terminated at time intervals of 2, 4, 6, 8, 10, 12, 14, and 16 min. At the end of each time interval, the reaction was supplemented with 100 μl of 34 % citric acid. The phosphate standard curve was compiled by plotting average absorbances on the *x*-axis and concentration of PO_4_ on the *y*-axis. All measurements were done in triplicate, and standard deviations were calculated.

### Protein extraction, quantification, and Western blot analysis

Sorghum protein extracts were prepared from 14-day-old treated (temperature-stressed) and untreated (control) plant materials. Protein extraction and quantification were performed as previously described (Ngara et al. 2012). For immunoblot assays, primary antibody (mouse anti-Hsp70/Hsc70 monoclonal antibodies, Product # SPA-820, Stressgen Bioreagents Corp., Victoria, Canada) and secondary antibody [goat anti-mouse IgG (H & L) horseradish peroxidase conjugate (Invitrogen Corp., Carlsbad, CA, USA)] were used as described previously (Ndimba et al. 2010).

### Quantitative real-time PCR

Quantitative real-time PCR was used to evaluate the expression profiles of Hsp70 in the leaves and stems of *S. bicolor*. This experiment was performed on a Light cycler® 480, based on the LightCycler® 480 SYBR Green I Master; 2X concentrate (Roche Applied Science, Germany). The reaction mixture contained 2 μl template cDNA, 10 μl 2X SYBR Green Master Mix, 1 μl of 10 μM of each primer, and 6 μl ddH_2_O. The reactions were subjected to 95 °C for 10 min, 45 cycles at 95 °C for 10 s, 60 °C for 10 s, and 72 °C for 20 s. A melting curve analysis was also performed using the default parameters on the LightCycler® 480. Each quantitative real-time PCR reaction was done in triplicate, and three non-template controls were included. The reference genes used included beta Actin, 18S ribosomal RNA (18S rRNA), ubiquitin (UBQ), and phosphoenolpyruvate carboxylase (PEPC). The primers used for real-time PCR are shown in Table 1. The expression levels of SbHsp70-1 gene were normalized to the reference genes and analyzed with the Light cycler® 480 SW (version 1.5) data analysis software. The expression was quantified by relative quantification method using a standard curve of serially diluted cDNA template. Figures were plotted on Microsoft Office Excel 2007. The experiment included three technical replicates, and it was repeated at least three times.

**Table 1:** Names of the genes and their accession numbers used for designing the primer used in the quantitative real-time PCR experiment

Reference Gene	Forward primer	Reverse primer	GenBank accession number	
Beta Actin	5′-CCT TAC CGA CTA CCT CAT-3′	5′-ATA GAT CCT TCC TAA TAT CCA-3′	AF369906.1	GI: 19851516
18S	5′-CCT TGA AAC AAC AAC GAT TA-3′	5′-CTG TGT CTA GGA CCA GTA-3′	XM_002455613	GI: 242053024
UBQ	5′-GCC AAG ATT CAG GAT AAG-3′	5′-TTG TAA TCA GCC AAT GTG-3′	XM_002452660	GI: 242062831
PEPC	5′-GAA GAA TAT CGG CAT CAA T-3′	5′-CTA TGT AAT ACT TGG TAA CTT TC-3′	XM_002438476	GI: 242096061
SbHsp70-1	5′-CTC CAT GAT CCT GAA CAA G-3′	5′-TGG GAG TCG TTG AAG TAG-3′	XM_002468052	GI: 242041404

### Cell viability under high temperature

To determine whether SbHsp70-1 confers heat tolerance *in vivo*, *E. coli* transformed with pET28a-SbHsp70-1 or empty pET28a vector (control) were subjected to heat stress, and their viability was determined. The overnight cultures were grown at 37 °C until cells reach an OD_600_ = 0.5, after which the culture was induced with 1 mM IPTG at 30 °C for 2 h. The cultures were diluted to equal absorbance of OD_600_ = 0.5 and diluted serially (1:10 and 1:100) with fresh LB. The effects of heat stress on the cells were examined by measuring the colonies formed after incubation and by the dot assay (Liu et al. 2009). Firstly, seven Eppendorf tubes containing 100 μl of the 1:100 diluted cultures were incubated at 50 °C for different time periods (0.5 to 3.5 h), and 50 μl from each time point was plated on LB plates (containing 25 μg/ml kanamycin and 1 mM IPTG), followed by overnight incubation at 37 °C. Cell viability was measured by counting the colonies formed after overnight incubation. These colonies were plotted as the percentage of colony forming units (CFUs) formed after heat treatment relative to the number of the CFUs formed from untreated cultures. Secondly, 5 μl of each sample (1:10 and 1:100) was spotted on LB plates (containing 25 μg/ml kanamycin and 1 mM IPTG) and incubated at 37 °C (control) and at 50 and 65 °C for 30 min followed by overnight incubation at 37 °C. The presence of SbHsp70-1 in the cultures was confirmed by immunoblot assays.

## Results

### Cloning of SbHsp70-1 gene

**Fig. 1:**
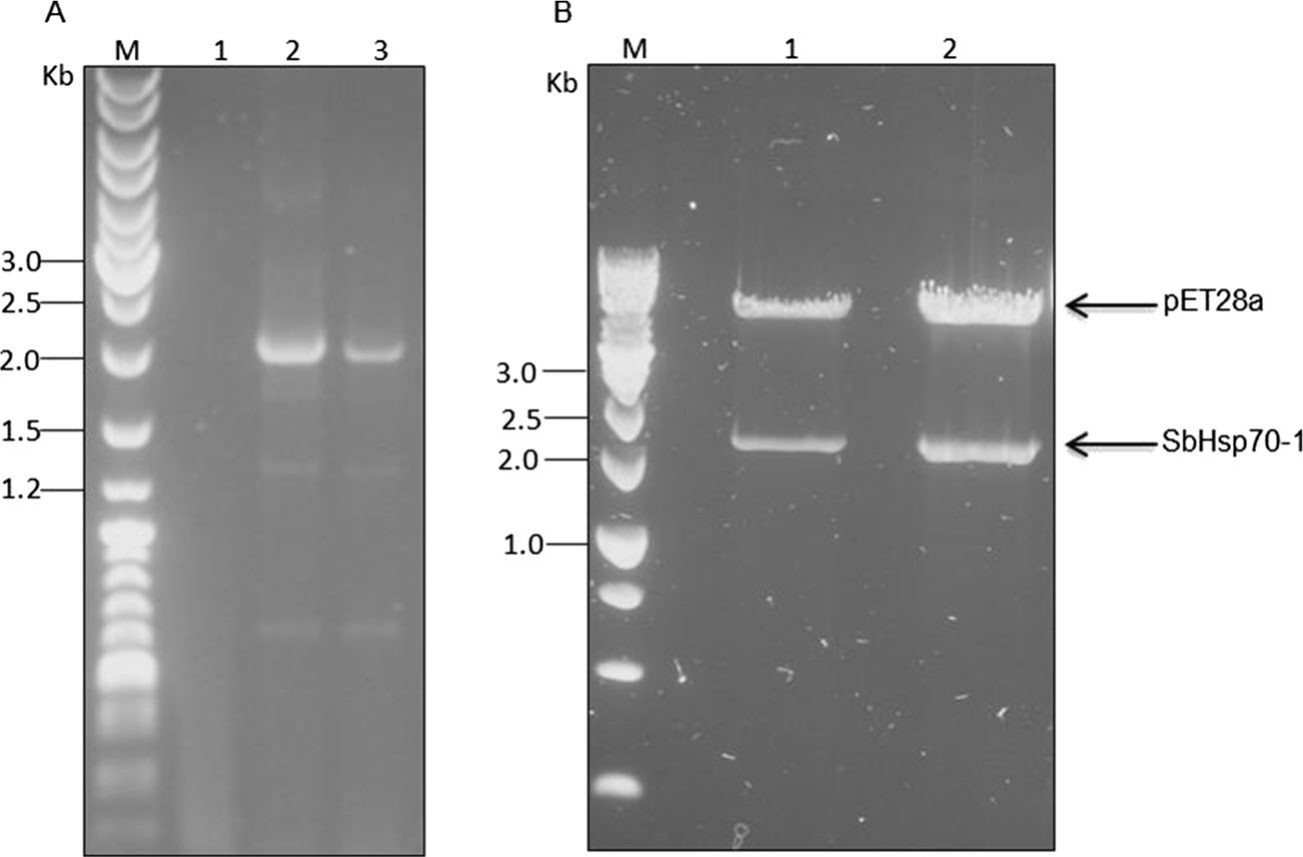
Agarose electrophoresis analysis of SbHsp70-1 fragments. **a** PCR amplification of SbHsp70-1 from sorghum cDNA. *Lane 1* corresponds to the negative control; *lanes 2* and *3* correspond to SbHsp70-1 fragments. **b** Restriction double digests of pET28a-SbHsp70-1 with *Bam*HI and *Xho*I. *Lanes 1* and *2*, digested plasmid; the bands correspond to the 5.3-kb pET28a and the 1.9-kb SbHsp70-1 as indicated by *arrows. M* refers to the molecular weight marker (O’GeneRuler DNA Ladder Mix #SM0331 and ThermoScientific GeneRuler 1-kb DNA Ladder #SM0311 in **a** and **b**, respectively)

Total RNA samples were isolated from 14-day-old sorghum whole seedlings and used to synthesize first-strand cDNA. Using gene-specific primers, cDNA fragments encoding the Hsp70 from *S. bicolor* were successfully amplified, indicated by a size of ~1950 bp (Fig. 1a). The recombinant clone was confirmed by PCR (results not shown) and double digestion of the pET28a-SbHsp70-1 construct with *Bam*HI and *Xho*I (Fig. 1b). DNA sequencing also confirmed the cloning, and hence the recombinant clone was designated as SbHsp70-1 (accession no. XM_002468052.1; GI: 242041404). The genomic DNA for SbHsp70-1 was amplified, and the amplicon size was observed to be the same as the cDNA fragment.

### Sequence analysis of SbHsp70-1

**Fig. 2:**
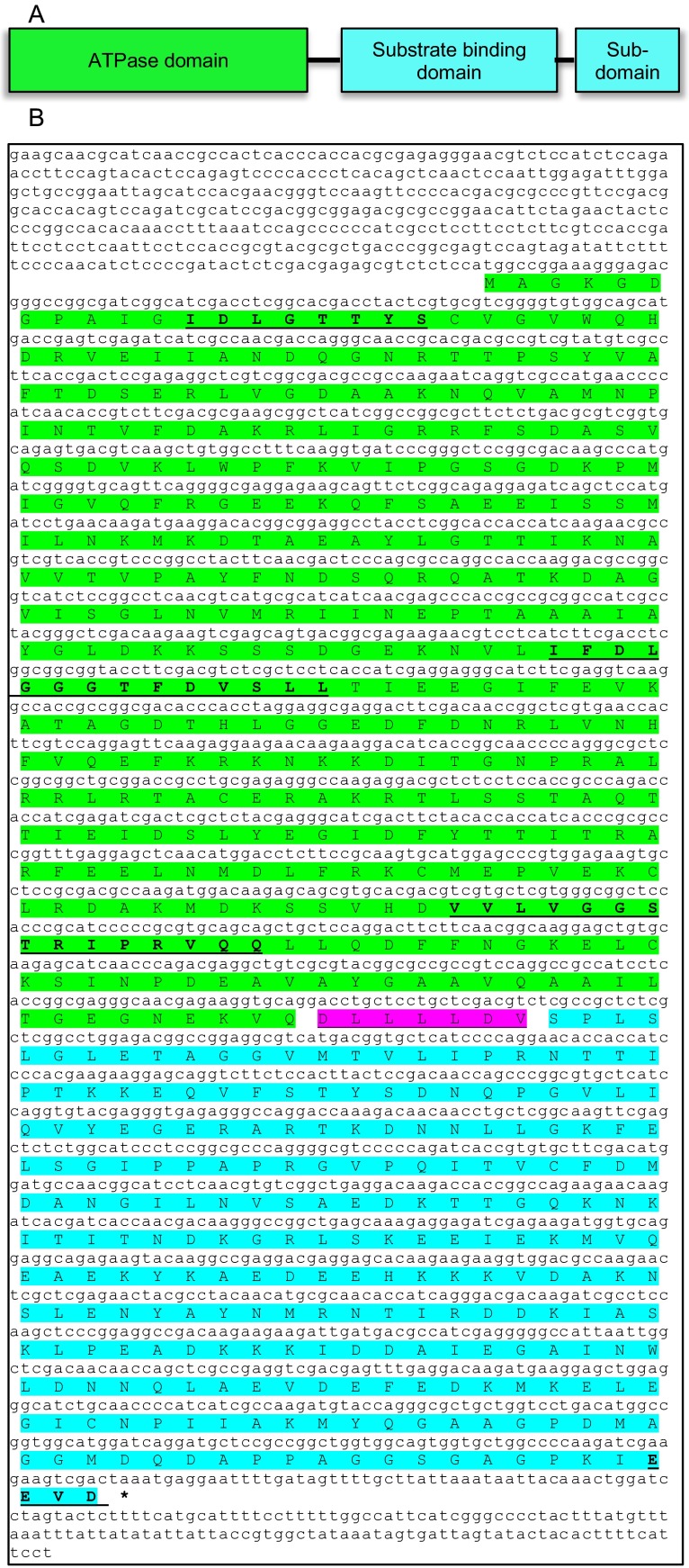
Sequence analysis of the SbHsp70-1. **a** The domain structure of SbHsp70-1; the *color coding* corresponds with the sequence (created in MS PowerPoint). **b** The nucleotide and the deduced amino acid sequence of SbHsp70-1 (sequence translated with Expasy tools and edited in MS word). The ORF of 1950 bp is shown starting with a start codon (*ATG*) and ended with a stop codon (*asterisk*). The conserved Hsp70 signature family motifs (1, 2 and 3), the ATP/GTP binding site and an EEVD motif are shown in *bold* and *underlined*. The linker joining the N-terminal ATPase domain and the C-terminal substrate binding domain is colored in *magenta*

**Fig. 3:**
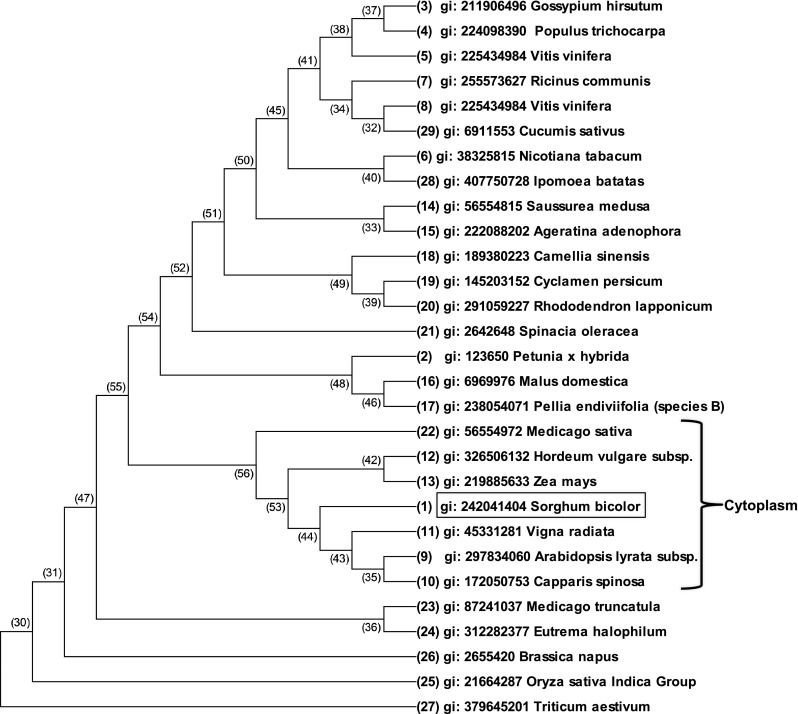
Phylogenetic tree (neighbor-joining) of the heat shock protein 70 from 29 plant species. The tree was computed using MEGA 6.0 program based on the amino acid sequences

The full-length cDNA of SbHsp70-1 was found to contain 2524 bp with an ORF of 1950 bp, which encodes a polypeptide of 649 amino acids (aa) with a molecular mass of 71.1 kDa and a pI of 5.08. SbHsp70-1 contains two functional domains, the N-terminal ATPase domain interconnected to a substrate binding domain by a linker (Fig. 2a). The conserved Hsp70 signature family motifs 1 (IDLGTTYS, 12–19 aa), 2 (IFDLGGGTFDVSLL, 203–217 aa), and 3 (VVLVGGSTRIPRVQQ, 340–354 aa), and an Actin-like ATP/GTP binding site (AEAYLGTT, 135–142 aa) are also found within the SbHsp70-1 sequence (Fig. 2b). Additionally, the protein was *in silico* predicted to be localized in the cytoplasm as characterized by the presence of an EEVD motif located at 646–649 aa, a characteristic of cytosolic Hsp70s. Blast searches of SbHsp70-1 with other plant Hsp70s revealed a strong amino acid sequence conservation (89–92 % identity). Phylogenetic analysis also showed that SbHsp70-1 shared high identity with those Hsp70s from 28 other plant species (Fig. 3). The tree also reveals that SbHsp70-1 is clustered among cytoplasmic localized Hsp70.

### Expression, purification, and ATPase activity assays of SbHsp70-1

**Fig. 4:**
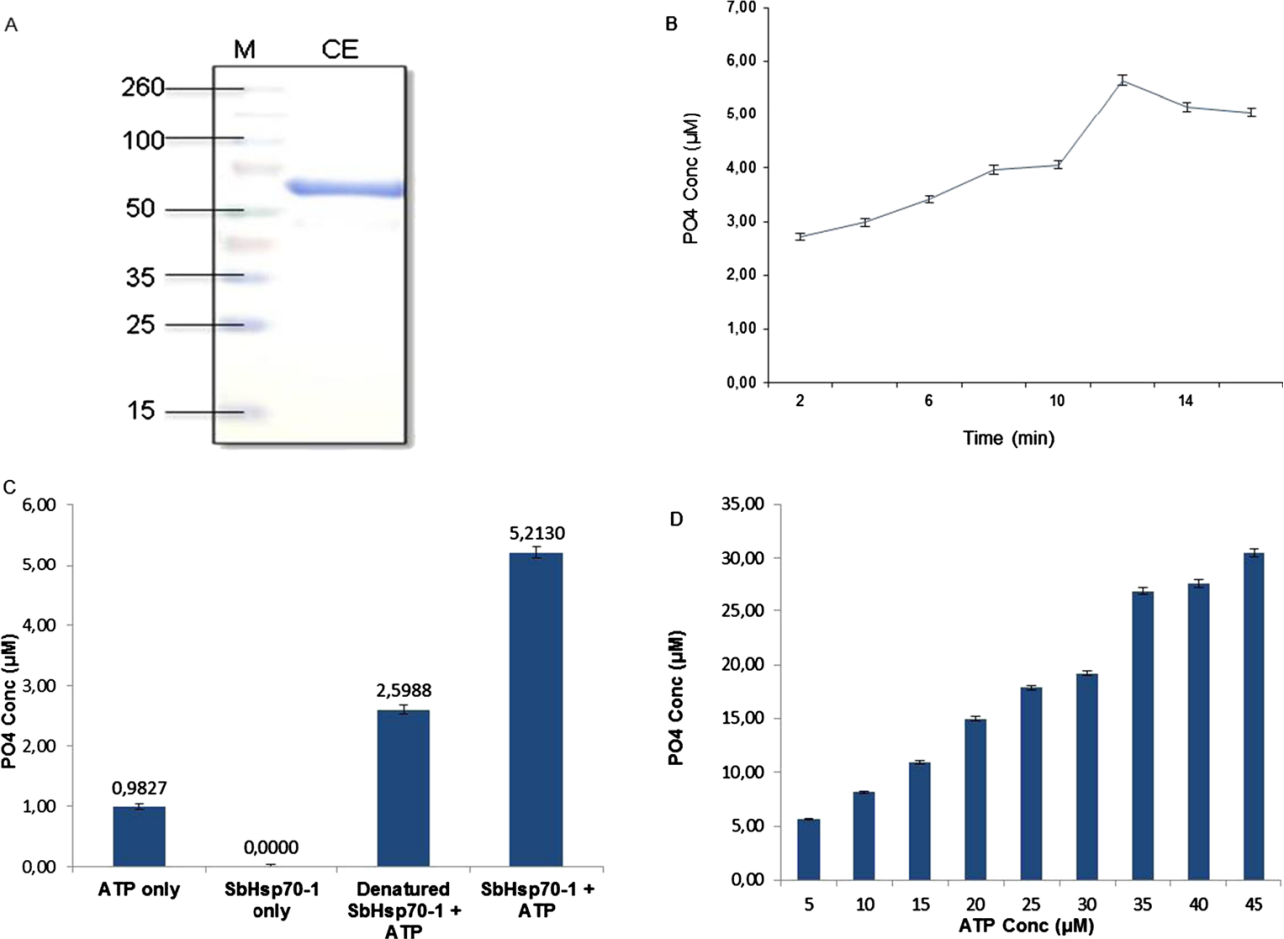
ATPase activity assays for recombinant SbHsp70-1 using a malachite green assay. **a** Eluted fractions from expressed recombinant SbHsp70-1 were concentrated and analysed on 12 % SDS-PAGE (*CE* concentrated elute). *M* refers to protein molecular weight marker (Spectra™ Multicolor Broad Range Protein Ladder, SM#1841). **b** The generated phosphate was measured in a time-dependent manner (2–14 min). **c** A fivefold increase in ATP hydrolysis in the presence of SbHsp70-1 (ATP + SbHsp70-1 full length) was observed compared to background (ATP alone). The denatured protein was also analysed and found to possess half the ATPase activity. **d** Release of phosphate measured at increasing substrate concentration (5 to 45 μM ATP). The *error bars* represent the standard deviations obtained for each experiment repeated three times

To investigate the biochemical role of SbHsp70-1, the gene was expressed in *E. coli* BL21-CodonPlus® cells as a fusion protein with a 6X Histidine-tag and purified by nickel affinity chromatography under native conditions. The expected protein band with the molecular weight of ∼72.1 kDa, which includes the calculated molecular weight of 71.1 kDa of SbHsp70-1, and a 6X Histidine-tag of 1 kDa was observed on the SDS PAGE. The full-length SbHsp70-1 was confirmed by anti-Hsp70/Hsc70 monoclonal antibodies using standard Western immunoblot assays (results not included). The purified protein was concentrated to 3 ml and resulted in an average yield of 29.13 mg/ml (Fig. 4a). Endogenous contamination with Dnak, the bacterial homologue of Hsp70, is a major concern when purifying Hsps due to its ability to bind to the expressed Hsps and co-purify with them (Nicoll et al. 2006). To eliminate the co-purification of endogenous Dnak, 10 mM ATP and 15 % glycerol were added to the wash steps.

The ATPase activity of SbHsp70-1 was assessed by measuring the concentrations of PO_4_ released from ATP using a modification of the malachite green colorimetric assay. As shown in Fig. 4b, the recombinant SbHsp70-1 was able to bind and hydrolyse ATP. ATP hydrolysis of the recombinant SbHsp70-1 and the subsequent release of PO_4_ increased gradually with time and eventually decreased after 12 min. To confirm the activity observed in Fig. 4b, control experiments including ATP in water (to determine the natural hydrolysis of ATP) and the recombinant protein without ATP were considered (Fig. 4c). The recombinant SbHsp70-1 in the presence of 5 mM ATP resulted in phosphate production of 5.2 μM. When using SbHsp70-1 which was denatured by heating at 95.5 °C for 20 min, the amount of phosphate decreased to 2.6 μM, representing a twofold reduction in ATPase activity. The recombinant SbHsp70-1 in the absence of ATP resulted in the release of 0.4 μM phosphate. In addition, ATP hydrolysis of SbHsp70-1 increased in a dose-dependent manner (Fig. 4d).

### Expression patterns of SbHsp70-1 in response to temperature stress

#### Western blot analysis of protein levels

**Fig. 5:**
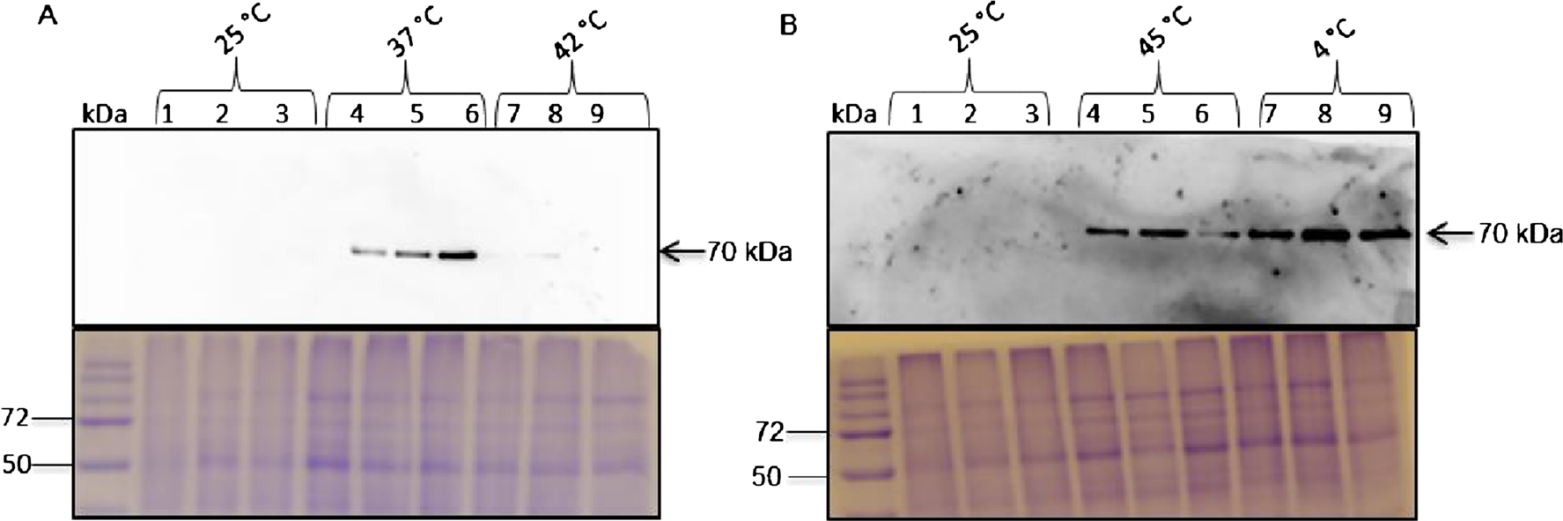
Analysis of the protein expression profiles of Hsp70 in *S. bicolor* under temperature stress. Fourteen-day-old sorghum seedlings were treated for 2 h under high and low temperatures. **a** Untreated (*lanes 1*–*3*), treated at 37 °C (*lanes 4*–*6*), treated at 42 °C (*lanes 7*–*9*). **b** Treated at 45 °C (*lanes 4*–*6*), treated at 4 °C (*lanes 7*–*9*). Total soluble proteins (20 μg) prepared from both untreated and treated seedlings were separated on 12 % SDS-PAGE and detected using anti-Hsp70/Hsc70, mAb

To study the protein expression levels of Hsp70 in sorghum under temperature stress, proteins from whole plant material were examined by SDS-PAGE and immunoblotting techniques. A 70-kDa protein detected by anti-Hsp70/Hsc70 monoclonal antibodies was observed in plant materials exposed to both high and low temperatures. Hsp70 protein was not expressed at the control temperature of 25 °C, and its abundance increased at 37 °C (Fig. 5a) and at 45 and 4 °C (Fig. 5b). Surprisingly, no induction of Hsp70 expression occurred at 42 °C (Fig. 5a).

#### Gene expression level analysis of SbHsp70-1

**Fig. 6:**
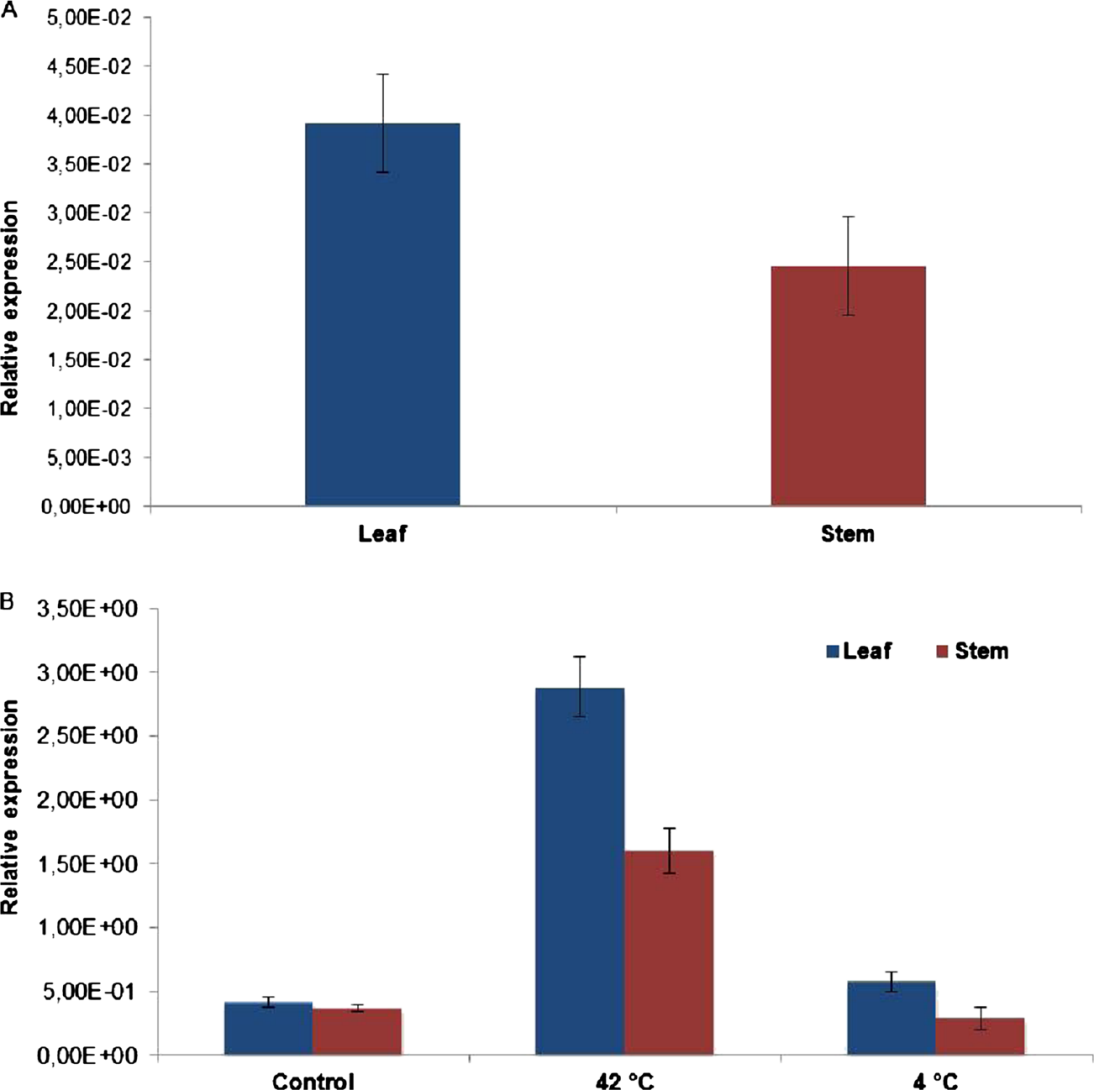
Expression patterns of SbHsp70-1 genes. **a** Analysis of the expression profiles of Hsp70 genes in sorghum leaf and stem tissues (control). **b** Expression of SbHsp70-1 under heat (42 °C) and cold (4 °C) stress. Expression data were normalized with reference genes used in this experiment. *Error bars* indicate the standard deviation

The mRNA expression level of SbHsp70-1 was determined in the leaves and stem tissues of *S. bicolor*. While mRNA transcripts were present in both tissues, the expression level was about twofold higher in the leaf than in the stem (Fig. 6a). Gene expression profiles of SbHsp70-1 during heat and cold shock were determined in sorghum seedlings exposed to 42 and 4 °C for 2 h, respectively. Transcript levels of SbHsp70-1 increased more than six- and fourfold in the leaf and stem upon heat shock, respectively. Upon cold shock, there was a slight increase in the leaf, but no significant change was observed in the stem (Fig. 6b).

### Thermotolerance assay of *BL21* cells expressing pET28a-SbHsp70-1

**Fig. 7:**
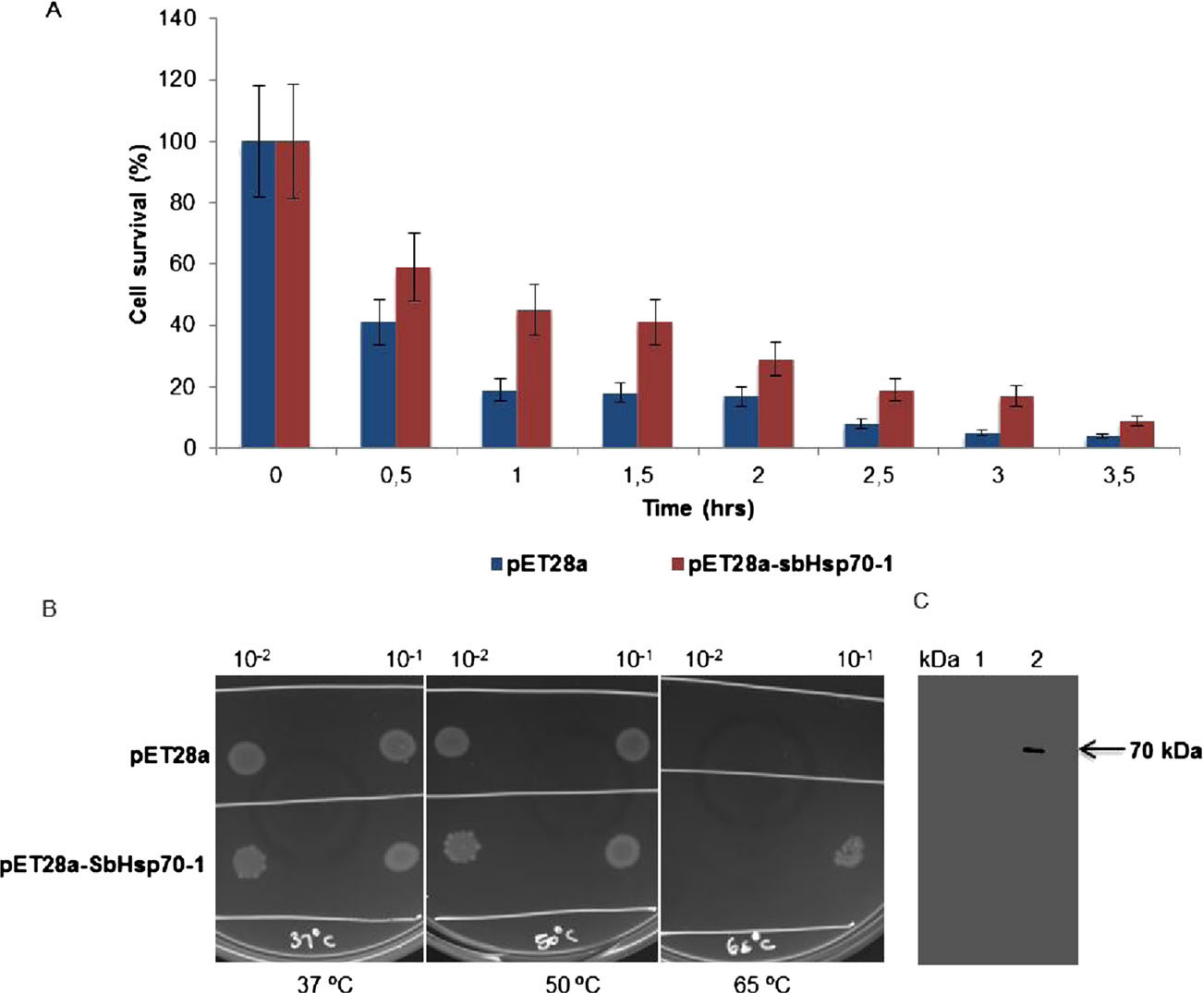
Effect of SbHsp70-1 on the viability of *E. coli* cells treated at high temperatures. **a** Cells transformed with pET28a vector (control) and pET28a-SbHsp70-1 were heat-shocked at 50 °C, and serial dilution of 1:100 was plated on LB plates. Data were collected for cells treated every 0.5 until 3.5 h. Viable cells were plotted as the percentage of the heat-treated CFUs relative to the number of untreated cells. The data represent the means of three independent experiments. *Error bars* represent standard errors. **b** Cells transformed with either pET28a or pET28a-SbHsp70-1 were treated at 37 °C (control) and at 50 and 65 °C for 30 min. **c** Immunoblot assay performed with monoclonal antibodies (anti-Hsp70/Hsc70, mAb) to confirm the presence of SbHsp70-1 at a molecular weight of 70 kDa in cells growing at 65 °C; *lane 1* cells carrying pET28a at 50 °C and *lane 2* cells transformed with pET28a-SbHsp70-1 at 65 °C

The molecular chaperone activity of SbHsp70-1 was investigated *in vivo* by overexpressing the fusion protein in *E. coli* cells and conducting thermotolerance assays. Serially diluted cultures transformed with pET28a or pET28a-SbHsp70-1 were treated at high temperatures of 37, 50, and 65 °C. Colonies were counted from the cultures treated at 50 °C for different time points of 0, 0.5, 1, 1.5, 2, 2.5, 3, and 3.5 h. Cells carrying the vector only were less viable compared to cells expressing SbHsp70-1, with the survival rate decreasing every 30 min. Cells carrying empty vector resulted in a 41 % survival compared to 59 % survival for the SbHsp70-1 expressing cells after 30 min, and after 2 h, cell viability from both cultures decreased significantly to below 20 % survival. It was observed that at 3.5 h, the viabilities of the cells expressing SbHsp70-1 were about twofold higher than the control cells (Fig. 7a). When the cells were spotted on the plates and treated at 37 and 50 °C, there was no distinct growth difference between the control cells and those expressing SbHsp70-1 for both temperatures (Fig. 7b). However, when cells were treated at an extreme temperature (65 °C), only the cells expressing SbHsp70-1 were able to grow (Fig. 7b), and no growth was observed for the control cells. Immunoblot analysis also confirmed that cells growing at 65 °C were protected by the presence of the fusion protein (Fig. 7c), indicating that SbHsp70-1 confers thermotolerance to *E. coli* cells.

## Discussion

The existence of Hsp70s in sorghum was demonstrated through analysis of protein expression profiles under salt stress (Ndimba et al. 2010; Ngara et al. 2012). However, the cDNA of sorghum Hsp70 has not been isolated and characterized. Previous mass spectrometry results of stressed sorghum seedlings indicated that sorghum Hsp70 had high sequence identity of 90.8 % with *Petunia* × *Hybrida*. To search for the Hsp70 gene in sorghum, the petunia Hsp70 sequence (accession no. PO9189.1; GI: 123650) was used to search the phytozyme (v9.1) in the *S. bicolor* (v1.4) database, and six sequences with the highest similarity were chosen for primer synthesis. In this study, two Hsp70 genes designated as SbHsp70-1 and SbHsp70-2 were successfully amplified from sorghum seedlings; however, only the SbHsp70-1 was cloned, sequenced, and characterized. Further studies are currently underway to sequence and characterize the SbHsp70-2 gene (XM_002442308.1; GI: 242083855).

The previously hypothetical gene now named SbHsp70-1 contains an ORF of 1950 bp, which encodes a protein of 649 aa. Sequence analysis of SbHsp70-1 protein indicated that it contains a highly conserved N-terminal ATPase domain and a C-terminal substrate-binding domain; however, there are variations in the C-terminal region. The Hsp70 signature motifs found in other characterized Hsp70s are also present in SbHsp70-1, suggesting that it might function as a molecular chaperone. SbHsp70-1 also contained a highly conserved EEVD protein motif located in the C-terminus. This motif facilitates the binding of Hsp70s with co-chaperones and is involved in the regulation of Hsp70s biochemical function (Johnson et al. 1998; Freeman et al. 1995). It has been predicted that Hsp70s which contain the EEVD motif at their C-terminal, a motif found in almost all studied Hsp70s, are localized in the cytoplasm (Lin et al. 2001) The predictions made by YLoc program and the presence of the EEVD motif strongly suggest that SbHsp70-1 is a cytosolic Hsp70 (Briesemeister et al. 2010). Sequence analysis also indicated that SbHsp70-1 contains multiple protein kinase receptor sites, which occur in eukaryotic proteins, suggesting that SbHsp70-1 might be involved in many regulatory reactions through phosphorylation and dephosphorylation (Kappes et al. 1993; Sherman and Goldberg 1993). In addition, phylogenetic analysis showed that SbHsp70-1 has high identity with Hsps from 28 other plant species; however, it forms a strong cluster with the cytosolic-localized Hsps and plant species classified as grain crops.

Expression of SbHsp70-1 in *E. coli* was successful, yielding a highly pure, soluble, and functional protein as confirmed by biochemical assays. Hsp70 possesses an ATPase activity, which is involved in many cellular roles such as prevention of protein aggregation and transport through its N-terminal ATPase domain (Pelham 1986; Chirico et al. 1988). ATPase activity of the recombinant SbHsp70-1 was examined and found to be time and dose dependent, releasing about 4.2 μM PO_4_ compared to ATP alone; thus, SbHsp70-1 functions as an ATPase. In this study, we investigated the expression levels of the Hsp70 protein/genes in sorghum using immunoblot and quantitative real-time PCR assays. In addition to the studies conducted by Ndimba et al. (2010) and Ngara et al. (2012), our present study investigated the expression of Hsp70s in *S. bicolor* under heat and cold shock treatments. Protein expression levels were investigated in whole plant material, and upon heat stress, Hsp70 protein levels increased as indicated by a strong signal on immunoblots detected at 37, 45, and 4 °C; however, the intensity decreased at 42 °C which was almost the same as the control plants (Fig. 5a). These results indicated the presence of Hsp70s in sorghum that could be induced by temperature stress. Gene expression was examined by quantifying the mRNA transcript levels of SbHsp70-1. Although its transcript was present in all the tested sorghum tissues, upon heat shock treatment its level was increased, while no change was observed under cold stress. These results indicated that the SbHsp70-1 gene is significantly induced by heat shock but slightly induced by cold, suggesting a role in heat tolerance. It is known that the inducible Hsp70 is expressed at low levels under normal conditions and increased significantly in response to various stresses, whereas the cognate form “Hsc70” is constitutively expressed under normal conditions, and their expression changes little or not at all under stress (Denlinger et al. 2001). Our results also indicated that SbHsp70-1 is expressed in both leaf and stem tissues but significantly increases upon exposure to heat shock. It is important to note that there were discrepancies between the protein and the mRNA transcripts levels. This might be because SbHsp70-1 induction at 37 °C is likely driven off by the existing mRNA, while at 42 °C, more mRNA is being transcribed for it to be available to make more protein which is required by the plant to cope with the increasing temperature stress. Upon cold shock, protein levels were increased; although the mRNA transcript level was increased in the leaf, there was no change in the stem. It is possible that since cold stress does not cause severe damage to the plant compared to heat stress, therefore when exposed to cold, Hsp70 is synthesized from the basal mRNA. Differential expression of the protein and the transcript under temperature stress suggests that SbHsp70-1 gene/proteins might be regulated both at a translational and transcriptional level. Further study is required and necessary to determine the precise mechanism of Hsp70 induction and function in sorghum under abiotic stresses.

Our expression profile experiments indicated that SbHsp70-1 transcripts are highly upregulated by heat stress. To investigate the possible chaperone role of the SbHsp70-1 gene, we tested its ability to protect *E. coli* cells against thermal stress. Many studies indicated that the expression of Hsps in the host cells displays positive protective roles against heat stress (Cha et al. 2012, 2009; Li et al. 2009). Overexpression of SbHsp70-1 in *E. coli* also enhanced the thermotolerance of bacterial cells (Fig. 6a); however, the viabilities of the cells decreased after 3.5 h, resulting in about a twofold increase in survival (6 %) compared to control cells. The same was observed in a study done by Cho and Bae (2007), in which the presence of NtHsp70-2 protected bacterial protein lysates from aggregation, thus enhancing thermotolerance to the cells. In addition, SbHsp70-1 was able to protect the bacterial cells from an extreme temperature (65 °C).

To our knowledge, there are no reports on the characterization of the Hsp70 genes from sorghum. Therefore, this study has paved a way for further research to elucidate the roles of sorghum Hsps in stress tolerance. Finally, this report demonstrates that SbHsp70-1 is a heat-inducible Hsp70 gene which was able to confer thermotolerance to *E. coli* cells, which may explain the ability of sorghum to survive under adverse environmental conditions.

## Electronic supplementary material

ESM 1 (XLSX 31 kb)

ESM 2 (XLSX 17 kb)

ESM 3 (XLSX 32 kb)
